# *Ligustrum × vicaryi* Fruit Polysaccharide Attenuates LPS/D-GalN-Induced Acute Liver Injury in Mice

**DOI:** 10.3390/ijms27198805

**Published:** 2026-10-01

**Authors:** Shu-Ling Liu, Hui Zhang, Jian-An Wang, Run-Dong Lv, Shao-Jian Li, Li-Tao Wang

**Affiliations:** 1 School of Pharmacy, Jining Medical University, Rizhao 276826, China; anansen@163.com (J.-A.W.); pharm_lv@163.com (R.-D.L.); 15263515685@163.com (S.-J.L.); 2 Institute of Immunology and Molecular Medicine, Jining Key Laboratory of Immunology, Jining Medical University, Jining 272067, China; zhanghuitjwh@hotmail.com

**Keywords:** *Ligustrum × vicaryi* L. fruit polysaccharide, acute liver injury, LPS/D-GalN, network pharmacology, ROC-PR

## Abstract

The dried fruits of *Ligustrum lucidum* Ait. are well-established in East Asian medicine for hepatoprotection. Given this ethnopharmacological precedent and their close taxonomic kinship, we hypothesized that the fruit polysaccharide of its horticultural congener, *Ligustrum × vicaryi* L. (LVFP), may confer analogous hepatic benefits. We assessed LVFP in a murine model of acute liver injury (ALI) induced by lipopolysaccharide and D-galactosamine (LPS/D-GalN) to test this hypothesis. LVFP pretreatment significantly mitigated hepatic pathological damage, normalized serum aminotransferases (AST, ALT), and reversed lipid peroxidation (reduced MDA, elevated SOD). Simultaneously, LVFP reduced pro-inflammatory cytokines, including interleukin-6 (IL-6) and tumor necrosis factor-alpha (TNF-α), and corrected lipid metabolic abnormalities (CHO, TG). To decode these phenotypic rescues, network pharmacology and molecular docking nominated a 10-gene hub signature (featuring KDR (binding energy = −8.65 kcal/mol), AKT1, GSK3B, and IL2). we further found that this signature—particularly KDR and PKM—robustly discriminates ALI from controls (AUC = 1.00; AUPR = 0.848), supporting its disease relevance. Collectively, these findings support our initial hypothesis that LVFP exerts multi-dimensional hepatoprotection.

## 1. Introduction

Acute liver injury (ALI), also called fulminant hepatic failure [1], represents a medical condition marked by a swift deterioration of hepatic function, resulting from a multitude of causative factors such as pharmaceutical toxicity, viral pathogens, autoimmune conditions, and ischemia–reperfusion injury [2]. The abrupt manifestation of liver failure can precipitate grave complications, including dysfunction of multiple organ systems, and is linked to considerable rates of morbidity and mortality. Presently, hepatoprotective approaches predominantly emphasize supportive care, owing to the absence of efficacious specific interventions for ALI. Consequently, the discovery and formulation of innovative therapies aimed at preserving hepatic function and alleviating hepatocyte injury are critically essential.

Plant polysaccharides, being natural products, have attracted significant attention, prompting the global scientific community to declare the 21st century as the age of polysaccharides [3,4]. Research has demonstrated that a variety of plant polysaccharides possess significant biological activity, including immunomodulatory [5,6,7], anti-tumor [8,9], hypoglycemic [10,11], hypolipidemic [12], anti-radiation, antibacterial and antiviral [13], and liver protection [14] activity. Both terrestrial and marine plant polysaccharides are recognized for their hepatoprotective capabilities. Terrestrial examples include the acidic polysaccharide derived from *Schisandra chinensis*, which protects against ethanol-induced liver damage in animal models and cellular systems by suppressing CYP2E1 protein expression and thereby diminishing oxidative stress-related injuries [15]. More directly relevant to acute liver injury (ALI), the polysaccharide from *Phaeoporus obliquus* demonstrated notable protection against ALI induced by carbon tetrachloride (CCl_4_) and alcohol in mice [16], while a polysaccharide isolated from the root of *Atractylodes macrocephala* protected against acute hepatic damage caused by CCl_4_ in a mouse model [17]. Marine polysaccharides likewise display well-documented hepatoprotective activities: fucoidan from brown algae protected against ConA-induced acute liver injury by inhibiting apoptosis [18]. Collectively, these studies establish that both terrestrial and marine plant polysaccharides can confer hepatoprotection, lending plausibility to the investigation of LVFP, whose specific effects and targets remain largely unexplored.

*Ligustrum × vicaryi* L., belonging to the Oleaceae family, is a hybrid species resulting from the cross-pollination of *Ligustrum ovalifolium* Hassk. var. aureo-mar ginatum and *Ligustrum vulgare* L. This species is frequently cultivated as an ornamental shrub, primarily valued for its distinctive golden foliage. Nevertheless, its pharmacological properties remain largely unexplored. Initial studies indicate that LVFP, a water-soluble polysaccharide isolated from *Ligustrum × vicaryi* L. with an approximate molecular weight of 88.9 kDa, has significant antioxidant, anti-inflammatory, and immune-modulatory properties [19], which are biological activities commonly associated with hepatoprotection. However, it must be clearly stated that, prior to this study, direct evidence for the hepatoprotective effect of LVFP against ALI was lacking. While related Ligustrum species (e.g., L. lucidum) have been reported to exhibit liver-protective effects in traditional medicine and modern pharmacological studies, the specific hepatoprotective potential of LVFP remained unknown and constituted the primary motivation for this investigation. Therefore, LVFP was selected for the present study to systematically evaluate its in vivo hepatoprotective effects and to elucidate its underlying molecular mechanisms.

**Figure 1:**
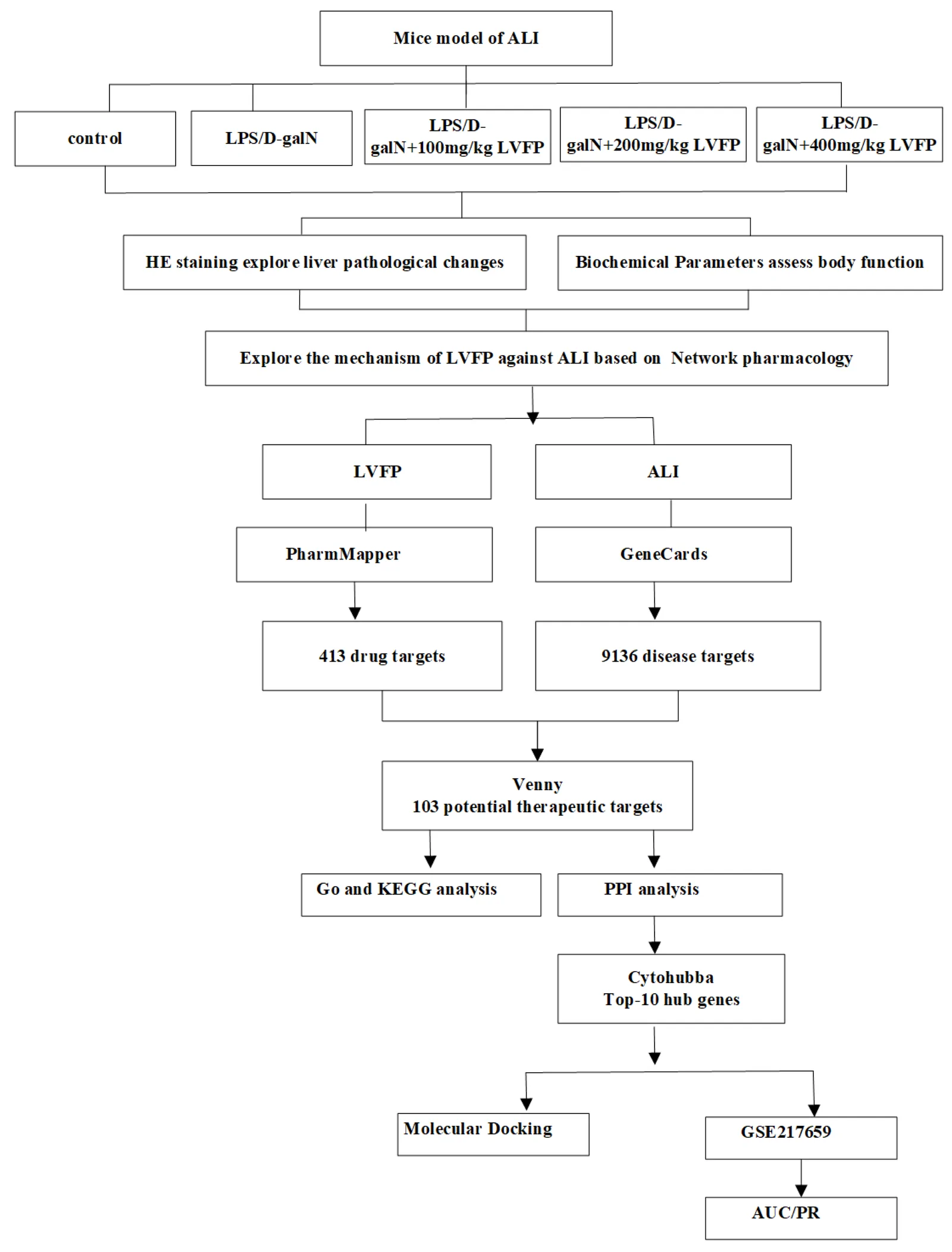
A comprehensive framework that outlines the manner in which LVFP addresses essential biological functions and mechanisms pertinent to the treatment of Acute Liver Injury (ALI). This approach is informed by findings from experimental assessments, network pharmacology, molecular docking studies and cross-cohort evaluation.

Network pharmacology represents an evolving discipline that merges systems biology with computational methodologies to elucidate the intricate interactions among drugs, targets, pathways, and diseases [20]. Therefore, building upon this genus-level rationale, the present study moves beyond anecdotal inference to systematically delineate LVFP’s in vivo hepatoprotection and decode its molecular nodes via integrated network pharmacology and external transcriptomic validation. We specifically focused on identifying hub genes capable of distinguishing disease states from controls, utilizing ROC and PR curve analyses to ensure robustness. This approach allowed us to nominate candidate targets for future mechanistic study. VEGFR2 (KDR), traditionally an endothelial angiogenic receptor, is also recognized for non-angiogenic roles in liver injury [21], and PKM has been implicated in the metabolic response of stressed hepatocytes [22]. Because our transcriptomic analysis identifies PKM at the gene level and no PKM2-specific validation was performed in this study, isoform-level mechanisms—including PKM2 dimer/tetramer balance, nuclear translocation, and HIF-1α/STAT3 signaling—are not elaborated here; a possible VEGFR2/PKM-centered mechanism is raised only as a working hypothesis for future investigation. The comprehensive framework of this investigation is illustrated in Figure 1.

## 2. Results

### 2.1. Effects of LVFP on Biochemical Parameters

**Figure 2:**
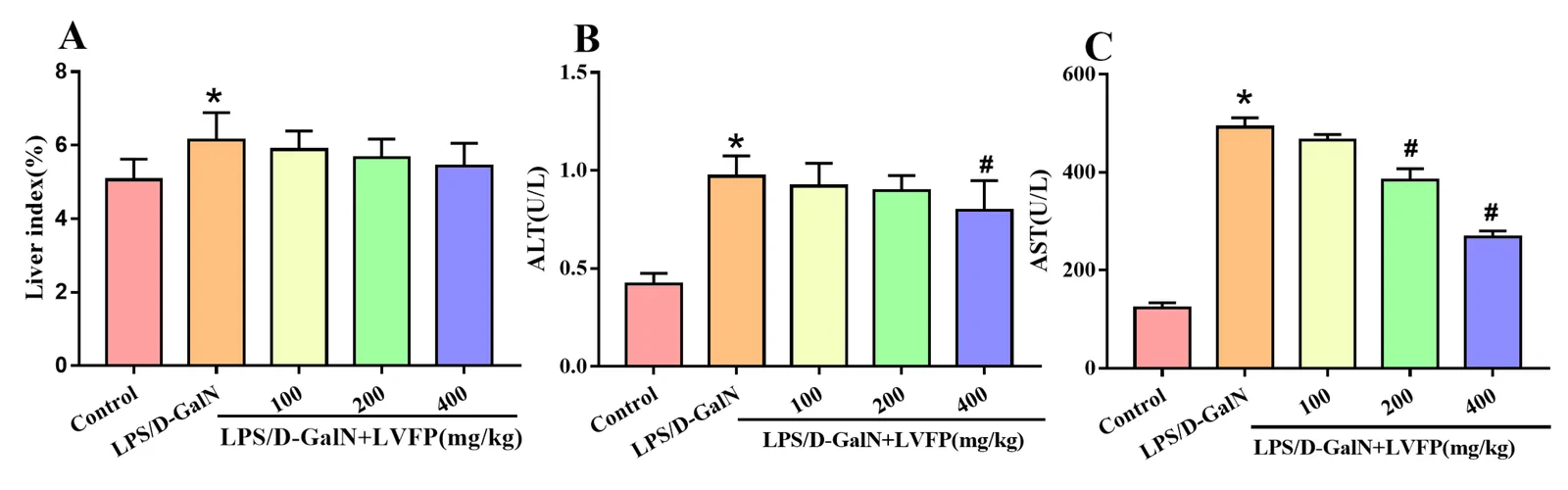
In a murine model of ALI induced by LPS/D-GalN, the impact of LVFP on the liver index (**A**), ALT activity (**B**), and serum AST concentration (**C**) was assessed. Mice with LPS/D-GalN-induced ALI were orally administered LVFP solution at doses of 100, 200, and 400 mg/kg per day for 7 days. Following the final treatment, all mice received an intraperitoneal administration of LPS at a concentration of 40 µg per kilogram of body weight and D-GalN at 600 mg/kg two hours later. Mice were weighed and blood samples collected via eyeball extraction within 24 h post-injection, prior to euthanasia. Serum samples were analyzed via spectrophotometry following the manufacturer’s guidelines. Results are presented as the mean with the standard deviation (n = 6). The bars designated distinct superscript letters denote statistically significant variation (*p* < 0.05). Symbols * *p* < 0.05 and ^#^ *p* < 0.05 represent comparisons with the control group and the LPS/D-GalN group, respectively.

This study aimed to clarify the hepatoprotective effects of LVFP on acute liver injury (ALI) caused by the combined administration of lipopolysaccharide (LPS) and D-galactosamine (D-GalN). This goal was accomplished through analyzing biochemical indicators and examining pathological changes in mouse models. Animal experiments demonstrated that administration of LPS/D-GalN markedly elevated circulating AST and ALT concentrations, confirming the onset of hepatic injury (refer to Figure 2). Higher-dose LVFP treatment produced a greater reduction in AST levels compared with the model group. Notably, the group receiving a high dose of LVFP showed the greatest reduction in ALT and AST levels, nearing normal physiological values (*p* < 0.05).

**Figure 3:**
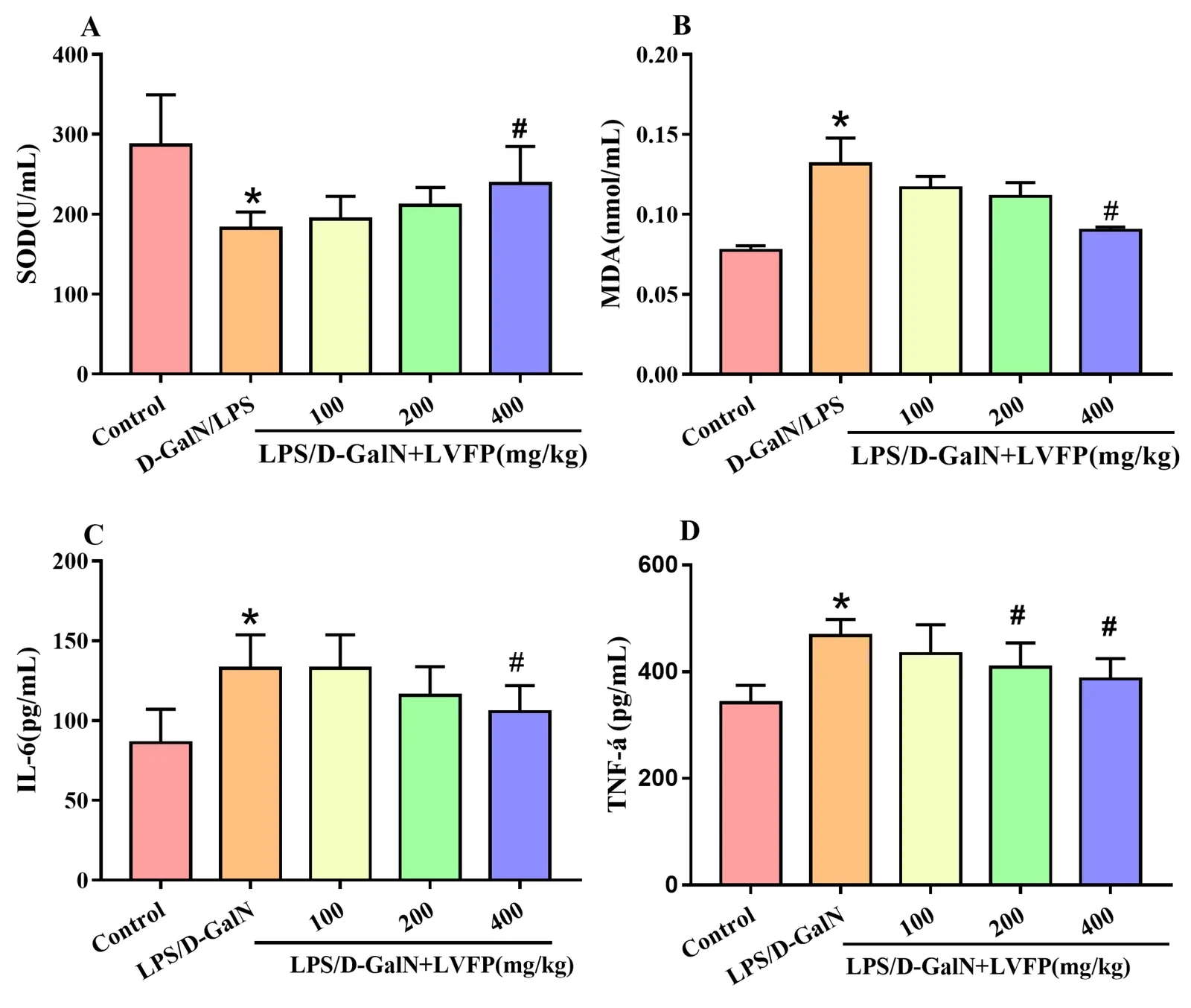
The study examined the effects of LVFP on liver tissue SOD, MDA, and serumIL-6, TNF-α levels in mice with LPS/D-GalN-induced acute liver injury. Biological samples were collected within 24 h post-treatment, liver tissue SOD, MDA, and serumIL-6, TNF-α levels were measured using specific assay kits. Data are expressed as mean ± SD (n = 6). Bars with distinct letters indicate statistically significant differences (*p* < 0.05). * indicates *p* < 0.05 compared to the control group, while ^#^ signifies *p* < 0.05 relative to the LPS/D-GalN group. (A) Liver tissue SOD activity; (B) liver tissue MDA content; (C) serum IL-6 level; (D) serum TNF-α level.

This study investigated the potential of LVFP to alleviate oxidative stress and inflammatory damage caused by LPS/D-GalN. Administering 400 mg/kg of LVFP significantly decreased MDA concentrations compared to the LPS/D-GalN group. The Exposure to LPS/D-GalN significantly reduced SOD levels relative to the control group. Importantly, the administration of 400 mg/kg LVFP significantly elevated SOD levels (Figure 3A). In contrast, as depicted in Figure 3B, the concentration of MDA demonstrated a significant rise upon exposure to LPS/D-GalN relative to the control group. The findings indicate that LVFP exhibits antioxidant characteristics that mitigate oxidative stress triggered by LPS/D-GalN. Upon stimulation with LPS, there was a release of pro-inflammatory cytokines accompanied by ROS, which may contribute to liver injury [23]. We evaluated the inflammatory response by measuring serum levels of IL-6 and TNF-α. Our study demonstrated that higher-dose LVFP administration led to greater reductions in serum IL-6 and TNF-α levels (see Figure 3C, D).

**Figure 4:**
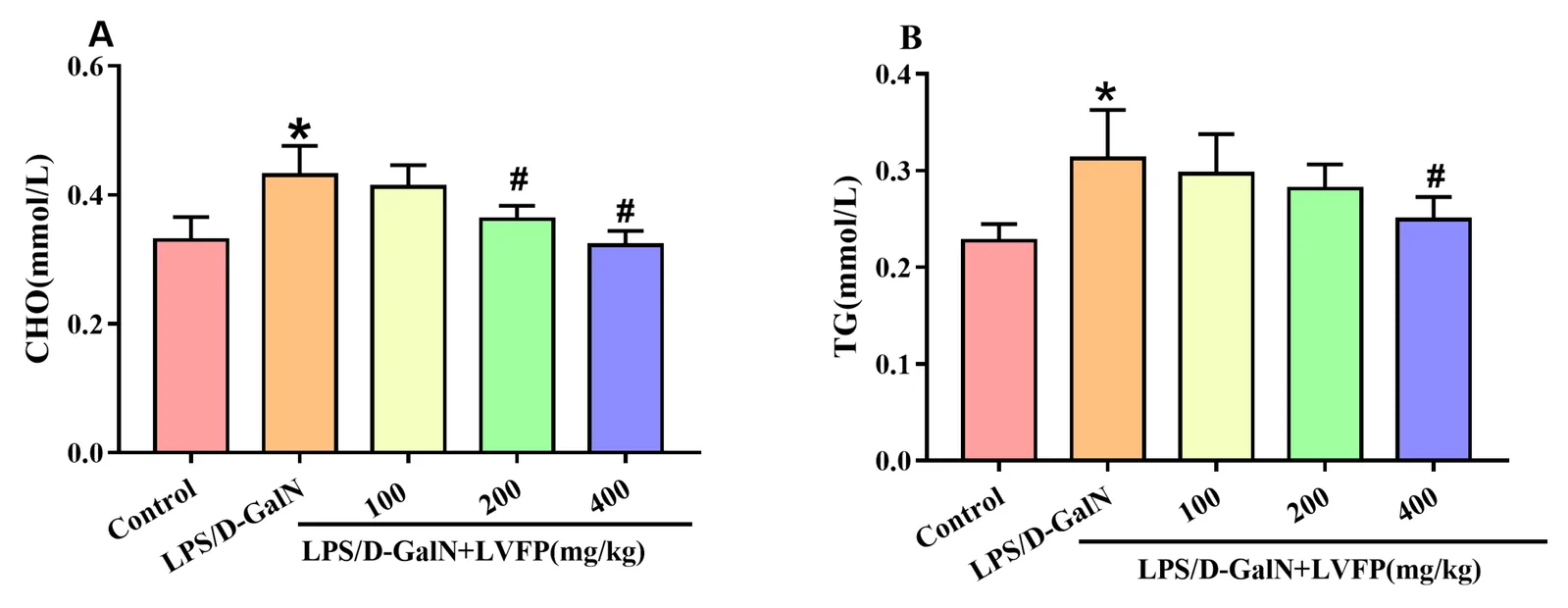
The effect of LVFP on serum CHO (**A**) and TG (**B**) levels was assessed using a mouse model of acute liver injury induced by LPS/D-GalN. Serum samples were collected, and CHO and TG levels were spectrophotometrically measured using specified kits within 24 h post-treatment. Data are presented as mean ± SD for n = 6. Bars labeled with distinct letters indicate statistically significant differences. Asterisks (* *p* < 0.05) denote comparisons to the control group, while hashtags (^#^ *p* < 0.05) indicate comparisons to the LPS/D-GalN treatment group.

Indicators of lipid metabolism comprise HDL, LDL, CHO, and TG. To assess the influence of LVFP on lipid metabolic processes, we quantified the serum concentrations of CHO and TG. The data presented in Figure 4 indicate a notable increase in CHO and TG levels following exposure to LPS/D-GalN when contrasted with the control group. LVFP administration significantly reduced CHO and TG levels compared with the LPS/D-GalN group, and higher-dose LVFP showed a greater effect. These results indicate that LVFP may exert a modulatory effect on lipid metabolic processes.

### 2.2. Histopathological Analysis

**Figure 5:**
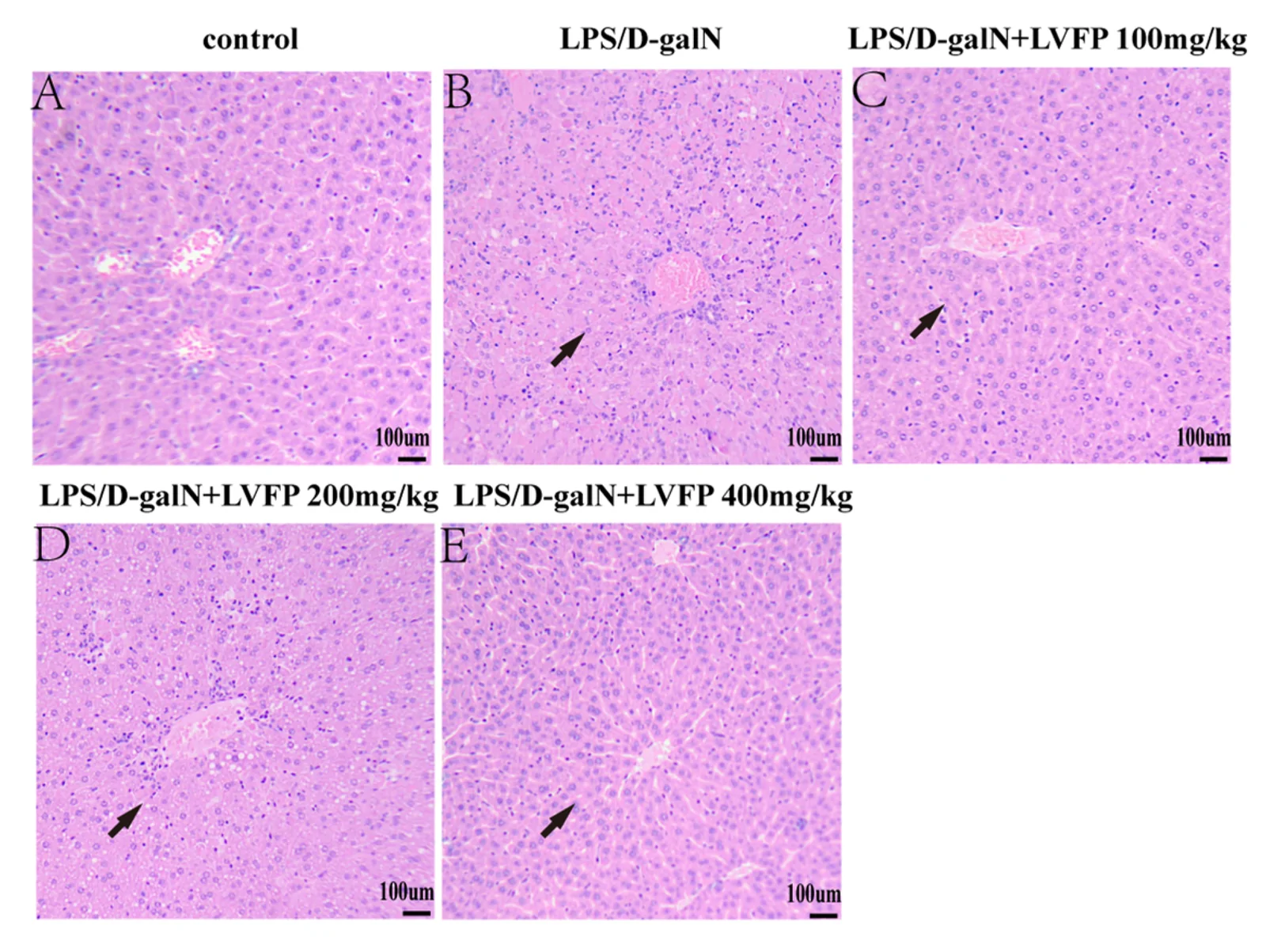
Histopathological evaluation of liver tissue sections stained with hematoxylin and eosin (H&E; scale bar = 100 μm). (**A**) Control group showing normal hepatic lobular architecture with orderly arranged hepatocytes around the central vein. (**B**) LPS/D-GalN-treated group showing severe hepatocellular injury; arrows indicate focal hepatocyte necrosis, disruption of hepatic lobular architecture, and inflammatory cell infiltration. (**C**) LPS/D-GalN + LVFP 100 mg/kg group; arrows indicate residual but attenuated hepatocyte necrosis and inflammatory cell infiltration. (**D**) LPS/D-GalN + LVFP 200 mg/kg group; arrows indicate further reduction in necrotic foci and inflammatory infiltration. (**E**) LPS/D-GalN + LVFP 400 mg/kg group; arrows indicate markedly preserved hepatic cord structure with minimal residual damage. All observations are representative of three independent experiments and were evaluated descriptively.

Histopathological analysis of liver specimens indicated extensive necrosis and fatty degeneration within the LPS/D-galN group, which was distinguished by inflammatory infiltrates and compromised hepatic architecture (Figure 5B). In contrast, the liver tissues of mice treated with LVFP showed qualitative improvements, characterized by a reduction in both necrosis and inflammation. The cohort administered a high dosage of LVFP exhibited histological features approaching those of the control group, as evidenced by a reduction in the quantity of inflammatory cells and the maintenance of hepatocyte structural integrity. These histological observations are qualitative and were not quantified by blinded scoring.

### 2.3. Integrative Bioinformatics Reveals Putative Targets Linking Efficacy to Mechanism

To explore the molecular basis underlying these phenotypic improvements, we subsequently conducted an integrative bioinformatics investigation including Network Pharmacology analysis and cross-cohort evaluation.

**Figure 6:**
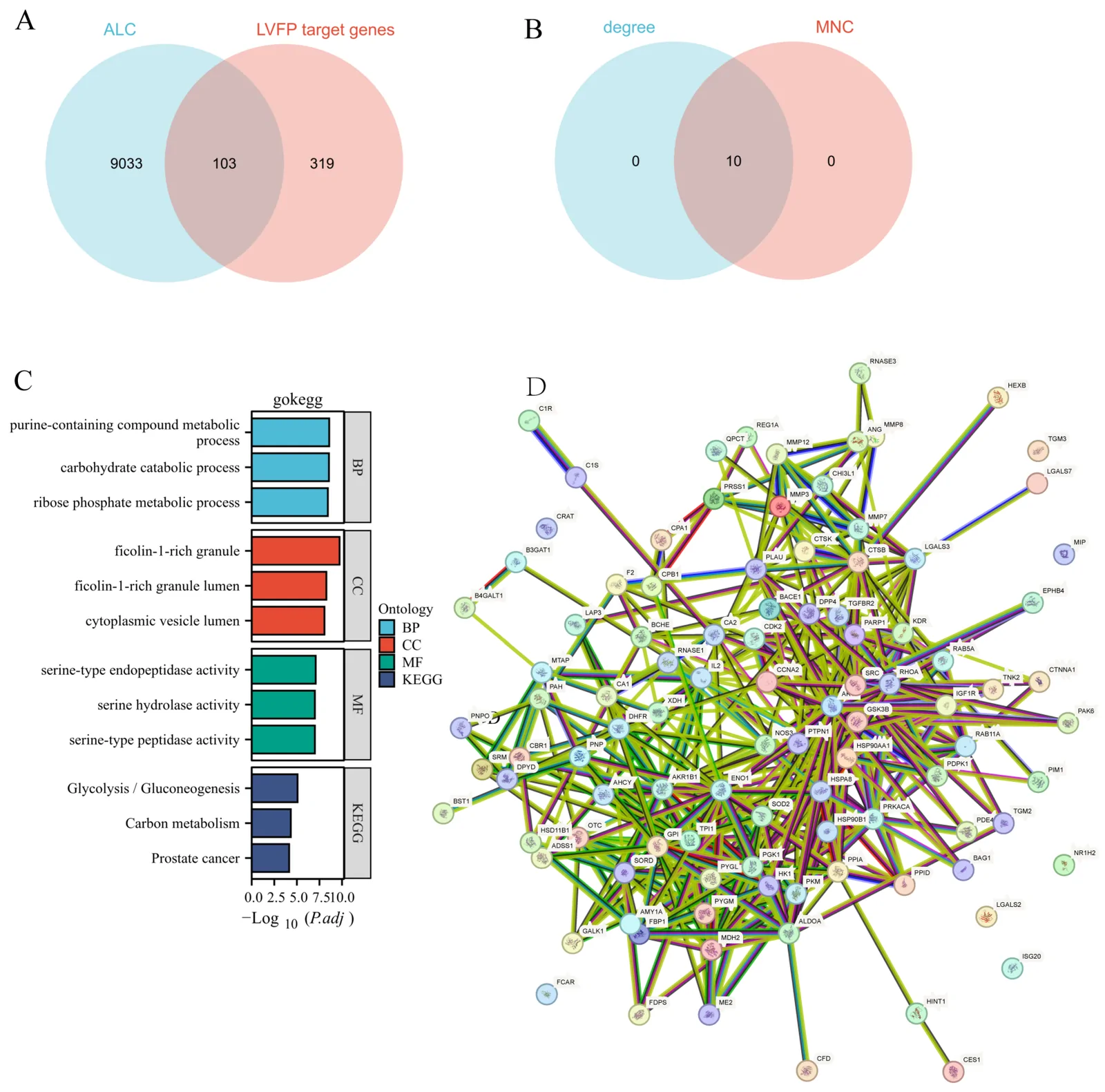
Target screening associated with LVFP in the treatment of ALI is illustrated as follows: (**A**) A Venn diagram depicting the possible 103 targets of LVFP in managing ALI; (**B**) A Venn diagram highlighting the 10 hub genes identified through degree and MNC analysis modules; (**C**) The significant GO and KEGG pathways that are enriched by the intersecting genes were analyzed using the Xiantao Website. (**D**) An analysis of the PPI network was performed using String (Version 12.0) to evaluate the 103 hepatoprotective targets associated with LVFP’s treatment of ALI.

The network pharmacology assessment unveiled a comprehensive total of 103 potential targets associated with LVFP that contribute to liver protection. The evaluation highlighted significant enrichment within several critical pathways, including glycolysis/gluconeogenesis, carbon metabolism, prostate cancer, all of which demonstrated a robust correlation with acute liver injury. The GO analysis indicated that these targets are mainly involved in biological processes like purine-containing compound metabolic process, carbohydrate catabolic process, and ribose phosphate metabolic process. A detailed overview of the principal targets and the enriched pathways is illustrated in Figure 6.

**Figure 7:**
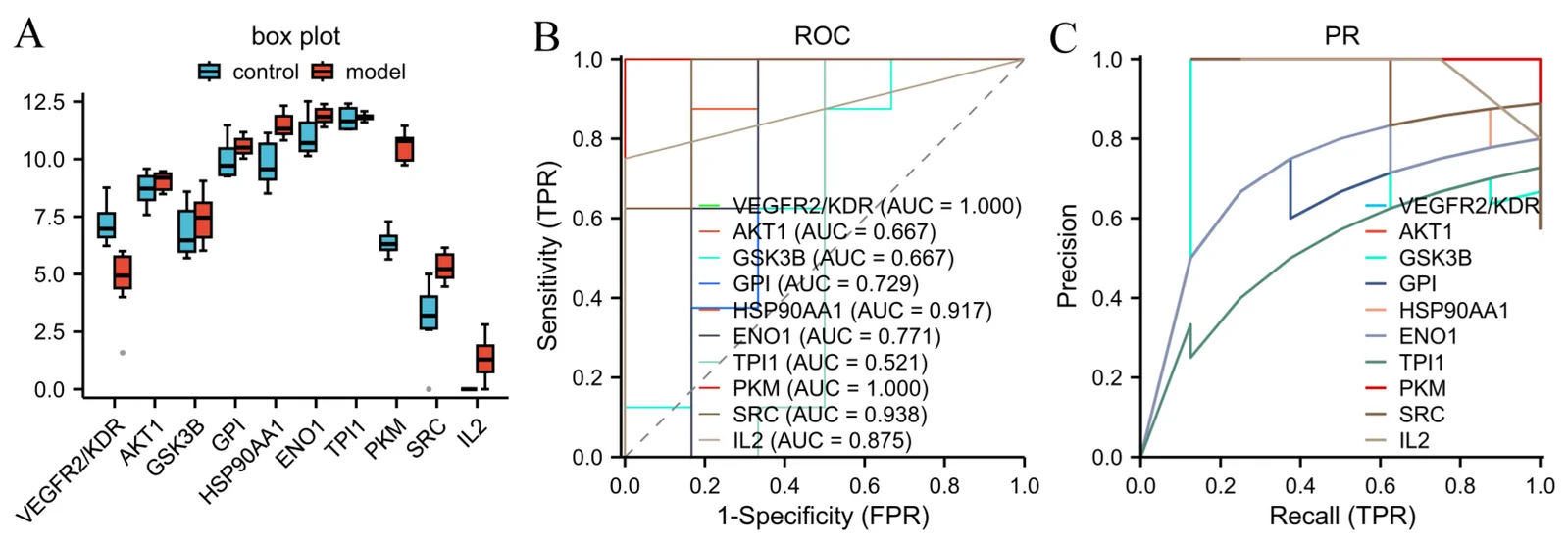
Cross-cohort performance evaluation of the 10 hub genes using the GSE217659 dataset. (**A**) 10-gene signature expression distribution in control and model groups. Box-plots show the expression levels of VEGFR2/KDR, AKT1, GSK3B, GPI, HSP90AA1, ENO1, TPI1, PKM, SRC and IL2 in control (blue) and model (red) samples. (**B**) ROC curves for the 10 computationally derived hub genes in the LPS/D-GalN-induced ALI cohort. VEGFR2/KDR and PKM exhibited excellent discriminative ability, with AUC = 1.00, indicating strong disease relevance in this ALI model, rather than regulation by LVFP. (**C**) PR curves for individual hub genes. Each curve reflects the trade-off between precision and recall for a single gene. AUPR values varied across candidates, with relatively higher performance observed for VEGFR2/KDR (0.848) and PKM (0.848). Other genes, including AKT1 (0.573), GSK3B (0.600), GPI (0.598), HSP90AA1 (0.798), ENO1 (0.632), TPI1 (0.489), SRC (0.804), and IL2 (0.725) showed heterogeneous discriminative capacity.

To validate the pathophysiological relevance of these computationally derived genes, we performed an independent cross-cohort analysis using the GEO dataset GSE217659, which was derived from the identical LPS/D-GalN-induced ALI model. Complementarily, the PR curve reinforced this finding, yielding an AUPR of 0.848 for VEGFR2/KDR and PKM (Figure 7C). Given the small external cohort (n = 14), we supplemented ROC-AUC with PR analysis to specifically assess performance on the ALI class. VEGFR2/KDR and PKM again showed superior discrimination (AUPR = 0.848 vs. baseline ≈0.57), whereas other hub genes performed poorly (AUPR 0.489–0.804; Figure 7C). The concordance between AUC and AUPR confirms the robustness of this prioritization against class imbalance. This high discriminative capacity suggests that VEGFR2 and PKM are not mere algorithmic artifacts but represent consistent and reliable biomarkers of ALI progression. Collectively, these data allow us to hypothesize that the observed in vivo efficacy of LVFP may be tentatively associated, pending experimental validation, via the modulation of this gene signature, particularly VEGFR2 and PKM.

### 2.4. Molecular Docking Studies

**Figure 8:**
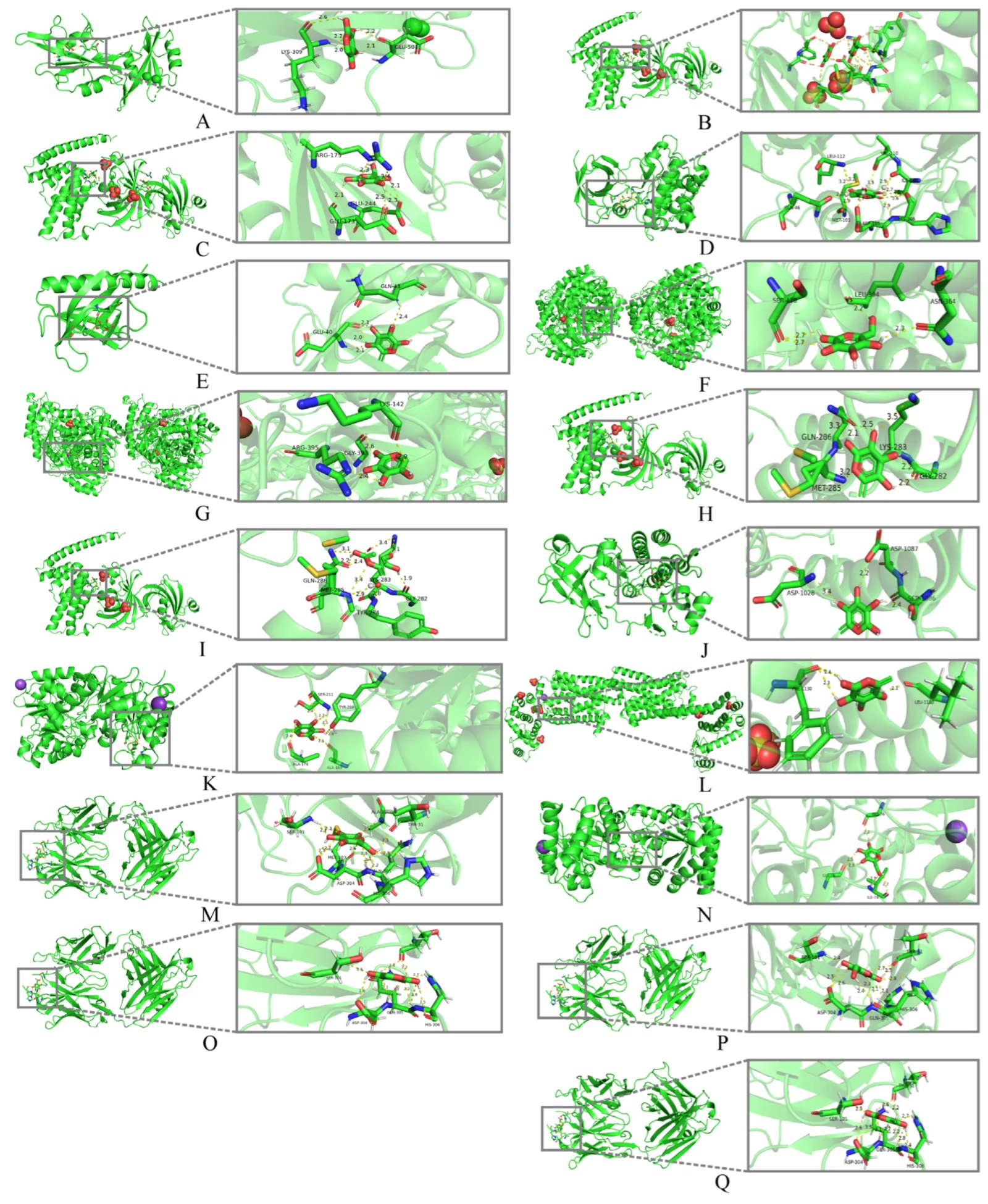
Molecular docking models of the monosaccharide constituents of LVFP with potential core anti-ALI targets. (**A**) SRC (1NZL)-Arabinose; (**B**) HSP90AA1 (5NJX)-Arabinose; (**C**) HSP90AA1 (5NJX)-Galactose; (**D**) GSK3β (2O5K)-Galactose; (**E**) AKT1 (1UNP)-Rhamnose; (**F**) ENO1 (7V67)-Glucose; (**G**) ENO1 (7V67)-Galactose; (**H**) HSP90AA1 (5NJX)-Rhamnose; (**I**) HSP90AA1 (5NJX)-Glucose; (**J**) VEGFR2 (3WZE)-Rhamnose; (**K**) TPI1 (4ZVJ)-Galactose; (**L**) PKM2 (7R6Y)-Rhamnose; (**M**) GPI (8BBH)-Galactose; (**N**) TPI1 (4ZVJ)-Galactose; (**O**) GPI (8BBH)-Arabinose; (**P**) GPI (8BBH)-Glucose; (**Q**) GPI (8BBH)-Rhamnose.

Molecular docking was performed using the constituent monosaccharides of LVFP (e.g., arabinose, rhamnose, galactose, glucose) rather than the intact LVFP polysaccharide due to computational limitations; these free monosaccharides are not equivalent to the native macromolecule, and we currently lack evidence that LVFP is converted to these free monosaccharides or reaches the proposed targets at biologically relevant levels. Molecular docking analyses indicated binding affinities between the LVFP constituents and ten hub targets. The computed binding affinity scores revealed that rhamnose, among the tested monosaccharides showed the strongest interaction with VEGFR2, achieving a docking score of −8.65 kcal/mol (Table 1), suggesting a plausible predicted interaction that warrants experimental verification. The docking inspections highlighted critical hydrogen bonds and hydrophobic interactions, both of which are vital for binding affinity. These results are presented only as a predictive, monomer-level exploration of potential ligand–receptor interactions and do not constitute direct evidence that intact LVFP binds VEGFR2, PKM/PKM2, AKT1, GSK3B, or other targets. The specific interaction modes for the three primary target proteins are depicted in Figure 8.

## 3. Discussion

The present study provides pharmacological evidence supporting the hepatoprotective efficacy of LVFP against LPS/D-GalN-induced ALI. Beyond the empirical observation that LVFP attenuates hepatic necrosis and systemic inflammation, our integrative bioinformatics approach offers a tentative mechanistic rationale for these effects. By intersecting LVFP-related targets with ALI-associated genes, we identified a core set of hub genes, notably VEGFR2/KDR and PKM. Their disease relevance was underscored by their discriminative power in an independent, identically induced ALI cohort GSE217659, where VEGFR2 and PKM achieved an AUC of 1.00 and an AUPR of 0.848; however, because this cohort contains ALI and control samples but no LVFP-treated samples, the ROC/PR analyses support only the disease relevance of these genes and do not demonstrate their regulation by LVFP. Furthermore, exploratory molecular docking yielded favorable predicted binding energies between free monosaccharides and these hubs at the monomer level only. Collectively, these convergent lines of evidence—ranging from phenotypic rescue to cross-cohort transcriptomic validation—allow us to raise a putative VEGFR2/PKM-related working hypothesis for future study, while noting that no PKM2-specific validation was performed in LVFP-treated animals and that any inference of VEGFR2/PKM2 modulation by LVFP remains speculative.

### 3.1. In Vivo Evidence of Hepatoprotection

The results obtained from animal experiments highlighted the protective role of LVFP, which was demonstrated by significant decreases in serum concentrations of ALT and AST after the administration of LVFP in models of ALI triggered by LPS/D-GalN. These enzymes are recognized as reliable indicators of hepatic damage; therefore, their normalization post-treatment strongly suggests a mitigation of hepatocellular injury.

Moreover, qualitative histopathological evaluations were consistent with the efficacy of LVFP, showing improvements in liver structure and a reduction in necrosis relative to the model group. As these observations were descriptive and not quantified by blinded scoring, they should be interpreted as qualitative support, suggesting that LVFP not only diminishes biochemical markers but also tends to improve the morphological impairments instigated by acute hepatotoxic agents.

The results indicate that LVFP provides hepatoprotective benefits by reducing oxidative stress, lowering pro-inflammatory cytokines, and managing cholesterol metabolism. A limitation is the lack of a positive control to benchmark LVFP’s efficacy against established hepatoprotective agents.

LVFP’s high arabinose and galactose content (~78%) likely contributes to its hepatoprotection, consistent with evidence linking RG-I-type polysaccharides rich in these sugars to antioxidant and immunomodulatory activities [23,24,25,26]. FT-IR spectroscopy confirms its acidic pectic nature (uronic acid bands at 1599, 1412 cm^−1^; Supplementary Figure S2). However, bioactivity is also governed by glycosidic linkages, substitution patterns, and molecular weight; full structural characterization (methylation, NMR) is required and noted as future work.

LVFP’s effects align with established hepatoprotectants: fucoidan inhibits apoptosis in ConA-induced ALI [18], while silibinin attenuates LPS/D-GalN injury via ASK1–p38 MAPK [27]. As both fucoidan and silibinin were tested in models relevant to our study, these comparisons strengthen the translational relevance of LVFP. Unlike small-molecule agents, LVFP likely acts via multiple mechanisms (antioxidant, anti-inflammatory, anti-apoptotic). Structure-activity studies are essential to define its active determinants.

### 3.2. Insights into Mechanisms Derived from Network Pharmacology

The analysis conducted through network pharmacology has revealed a wide array of potential targets related to LVFP, indicating that its effects are likely the result of multiple pharmacological actions rather than the influence of a single target. Prominent pathways associated with LVFP include glycolysis/gluconeogenesis, prostate cancer pathways, and the metabolism of fructose and mannose. These pathways are vital for processes such as pyruvate metabolism, the modulation of oxidative stress responses, and the regulation of cell death triggered by oxidative stress, aligning with findings from animal studies. The influence of these pathways suggests that LVFP may function as a multi-target hepatoprotective agent, enhancing hepatocyte viability while concurrently mitigating inflammation and apoptosis in the context of hepatic injury. Additionally, the detection of monosaccharides in LVFP, confirmed by our HPGPC analysis, reinforces the idea of synergistic effects. Each monosaccharide may offer distinct contributions to the overall hepatoprotective effects, thereby augmenting the hepatoprotective efficacy of LVFP in comparison to individual compounds.

In this multi-target landscape, KDR (VEGFR2) and PKM are biologically plausible candidate anchors, given their documented involvement in liver injury responses and metabolic regulation. However, it must be emphasized that the transcriptomic analysis identifies PKM at the gene level, and no PKM2-specific assays were performed in LVFP-treated animals in this study. Defining whether and how LVFP modulates these nodes will require direct protein-level validation.

### 3.3. Molecular Docking: In Silico Prediction of Binding Affinity

Molecular docking provided exploratory, monomer-level predictions of the possible binding interactions between the free monosaccharide constituents of LVFP and candidate liver-associated targets, including AKT1. These predictions do not demonstrate direct interaction between intact LVFP and these proteins. AKT1 is recognized for its function in enhancing cell survival and suppressing apoptosis; whether such a protective signaling cascade is engaged by LVFP in vivo remains to be experimentally tested.

These observations are consistent with studies indicating that polysaccharides derived from various herbal sources exhibit comparable protective mechanisms. The capacity of LVFP to interact with multiple proteins is proposed only as a hypothesis, requiring direct experimental validation in vivo.

Among hub targets, VEGFR2 (KDR) and PKM are prominent in ALI pathophysiology. Docking of free monosaccharides to VEGFR2 (3WZE) and PKM2 (7R6Y) yielded favorable predicted energies, with the VEGFR2–rhamnose pair showing the lowest predicted energy (−8.65 kcal/mol). These results are exploratory predictions at the monosaccharide level only: they do not establish direct binding of intact LVFP to VEGFR2 or PKM2, nor modulation of VEGFR2/PKM2 signaling in vivo. Any inference that such interactions interfere with PKM2 dimer–tetramer equilibrium or VEGF-driven activation remains hypothetical and requires direct binding and functional validation.

### 3.4. Implications for Clinical Application

The results of this investigation indicate that LVFP exerts a hepatoprotective effect in a preventive mouse model of LPS/D-GalN-induced acute liver injury. Because the present study did not include clinical data, pharmacokinetic analysis, or sufficient translational validation, no clinical application of LVFP can be proposed at this stage. Further investigations, including safety profiling, bioavailability, and metabolic studies, are required before any translational consideration.

### 3.5. Limitations and Perspectives

While our study robustly confirms the hepatoprotective efficacy of LVFP in a well-established LPS/D-GalN model, and bioinformatics analyses provide a compelling mechanistic hypothesis, certain limitations should be acknowledged. Principally, the regulatory effects on the prioritized hub genes and pathways were inferred via ROC/PR evaluations of public datasets and molecular docking, rather than directly measured in our animal cohorts such as via qPCR or Western blot.

Therefore, the current work serves as a foundational “efficacy-plus-hypothesis” study. The high discriminative power (AUPR) of our identified gene signature strongly suggests biological plausibility; nonetheless, direct genetic or pharmacological interventions (knockdown/overexpression) and tissue-specific validation of these targets are warranted in future investigations to definitively prove causality. Additionally, although sample size limited advanced regression modeling, our dual-curve approach maximized the informational yield from the available data.

This study’s limitations require careful consideration, particularly its reliance on a single ALI model induced by LPS and D-GalN, which may not fully represent the complexities of human ALI. Furthermore, the mechanistic understanding attained from bioinformatics evaluations and molecular docking necessitates validation through more comprehensive in vivo investigations, given that the current outcomes are predominantly correlative in nature. It is advisable that future research endeavors should investigate the hepatoprotective potential of LVFP across diverse injury models, thereby enhancing the translational implications of these findings.

## 4. Materials and Methods

### 4.1. Preparation of Polysaccharide Extracts and Characterization

LVFP samples were obtained from a botanical garden situated within Jining Medical University in Rizhao, Shandong Province, China. The plant species was identified by Jianan Wang, a medical botanist associated with the School of Pharmacy at Jining Medical University. A voucher specimen has been archived at the herbarium of the aforementioned institution. The extraction process of LVFP was performed in accordance with the established methodologies previously described [19]. Briefly, LVFP was extracted from 500 g dried fruit powder via acetone defatting, water ultrasound extraction, ethanol precipitation, de-pigmentation, Sevag deproteinization, and lyophilization (yield: 1.6%, 7.9 g). The total carbohydrate content was determined by anthrone–sulfuric acid method using glucose as the standard (y = 7.67x − 0.0181, R^2^ = 0.9997, Figure S1). The carbohydrate content was 80%. As a continuation of our group’s previous work [19], LVFP was initially characterized using HPAEC-PAD and FT-IR spectroscopy. In this study, we supplemented the analysis with additional FT-IR detection, the results of which were consistent with previous data, confirming the identity of the polysaccharide component. LVFP was analyzed by FT-IR (IRTracer-100, Shimadzu, Kyoto, Japan; KBr pellet, 4000–500 cm^−1^; Supplementary Figure S2).

### 4.2. Animal Experiments

**Figure 9:**
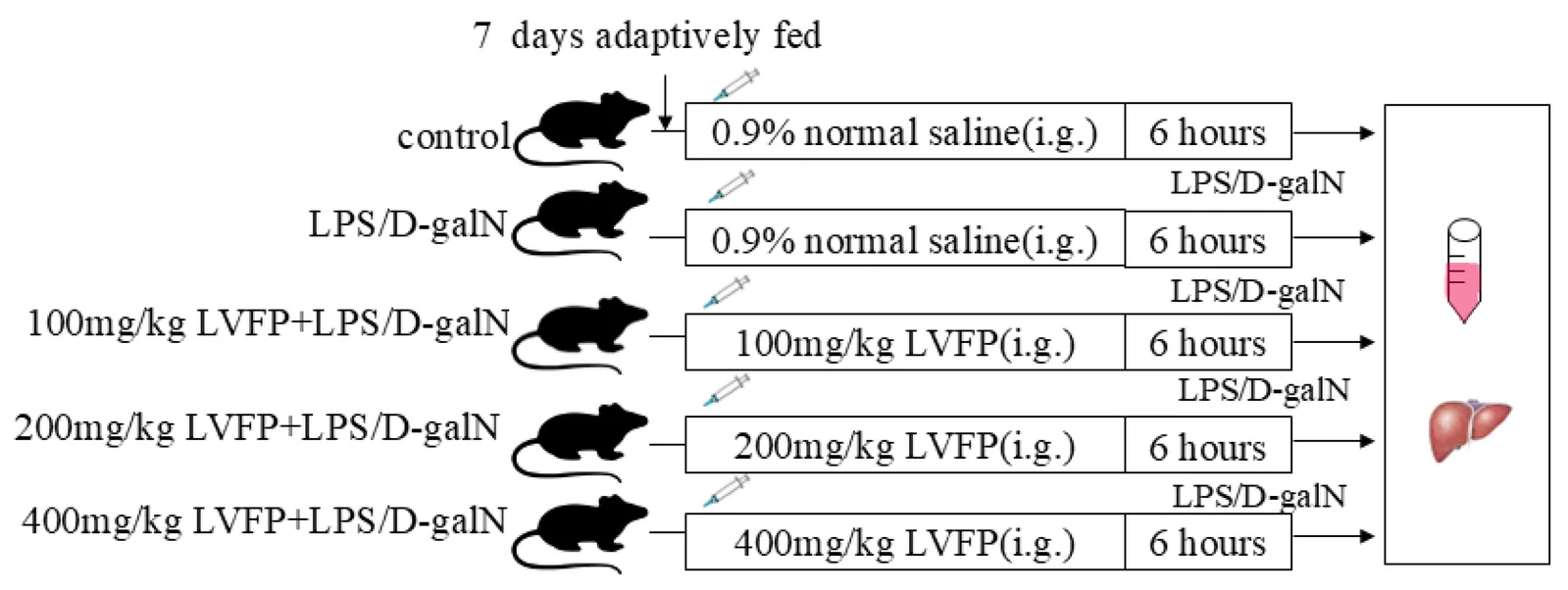
The schematic diagram presented outlines the experimental design involving animals utilized in the current research.

C57BL/6J mice, weighing 20 to 22 g, were sourced from the Animal Center of Jinan Pengyue Laboratory Animal Breeding Co., Ltd., Jinan, China (Experimental Animal License SCXK-Shandong-2022-0006).The experiments adhered to the guidelines set by the Institutional Animal Care Committee of Jining Medical University, China (SYXK-Shandong Province-2024-0004).The research was approved by the Jining Medical University Animal Care and Use Ethics Committee (Authorization No.JNMC-2023-DW-131; approved on 21 November 2023).Mice were kept in a controlled environment with a 12-h light/dark cycle and had ad libitum access to standard rodent chow and water. Prior to initiating the experimental procedures, the animals underwent a one-week acclimatization phase. Acute liver injury was induced by administering an intraperitoneal injection of lipopolysaccharide (LPS) at 40 µg/kg and D-Galactosamine (D-GalN) at 600 mg/kg, two hours after the final injection. Control mice received a comparable volume of saline. Our previous studies indicated that LVFP (400 mg/kg/day) exhibited both hepatoprotective efficacy and safety in treatment cohorts, which were administered via intraperitoneal injection over a period of 7 days. Before inducing ALI, the experimental mice were randomly divided into five groups, each containing six animals: a control group receiving saline, a model group also given saline, and three treatment groups administered LVFP at low (100 mg/kg), medium (200 mg/kg), and high (400 mg/kg) doses. LVFP was administered through gavage once daily for 7 consecutive days. After the treatment period, blood samples were collected through ocular puncture for serum biochemical analysis to evaluate liver function by measuring ALT and AST levels. Upon euthanasia, the mice were anesthetized using inhaled carbon dioxide prior to decapitation, and their bodies were frozen and sent to a specialized animal treatment site. The liver tissues were collected for both histological evaluation and molecular analysis. Tissues were preserved in 10% neutral-buffered formalin, embedded in paraffin wax, and sectioned to a 5 µm thickness. Standard histological techniques, including hematoxylin and eosin (H&E) staining, were used to evaluate histopathological changes. The prepared slides were examined and photographed using a light microscope (Olympus, Tokyo, Japan) at a magnification of 100x, with representative images presented in the results. Figure 9 depicts the design of the animal experiments conducted in the current study.

### 4.3. Molecular Analysis

Serum samples were collected to measure key inflammatory cytokines, TNF-α (SEKM-0034) and IL-6 (SEKM-0007), using ELISA kits from Beijing Solarbio Science & Technology Co., Ltd., Beijing, China. Lipid metabolism markers, CHO(A111-1-1) and TG(A110-1-1), were quantified using detection kits from Nanjing Jiancheng Bioengineering Institute, Nanjing, China. Subsequently 40% carbon dioxide euthanasia, liver tissue was excised from the mouse. The tissue was homogenized and subjected to centrifugation to obtain the supernatant. The supernatant was analyzed for the levels of superoxide dismutase (SOD, A001-3-2) and malondialdehyde (MDA, A003-1-2) in the liver tissue homogenate assay kits.

### 4.4. Bioinformatics Prioritization and Cross-Cohort Analysis

In order to clarify the possible mechanisms through which LVFP may exert hepatoprotective effects against ALI, we adopted a network pharmacology methodology characterized by several systematic steps. Initial analyses using FT-IR spectroscopy, HPIC, and HPGFC identified that LVFP contains arabinose, rhamnose, galactose, and glucose in a molar ratio of 7.55:1.79:4.58:1.54, with a molecular weight of 88,949 Da [19]. LVFP monosaccharides were analyzed in TCMSP for chemical structures, DL (≥0.18), and OB (≥30%). Targets were predicted by PharmMapper (version 2017) and supplemented with GeneCards (version 5.26.0). PPI networks (STRING, confidence ≥ 0.400, min. 10 interactions) were visualized in Cytoscape (3.10.3), and hub genes were identified via degree, betweenness, and closeness centrality. Given the exploratory nature of this study and the absence of direct genetic assays in the current cohort, we utilized an independent GEO dataset GSE217659 derived from the same LPS/D-GalN ALI model. ROC and PR curve analyses were performed to evaluate the discriminative power of these candidates, thereby generating a prioritized list of targets (e.g., VEGFR2, PKM) for future confirmatory studies. Due to the limited size of GSE217659 (n = 14), ROC–AUC was supplemented with PR analysis, which is more sensitive to classifier performance on the ALI-positive class in small cohorts [28]. ROC–AUC provided global ranking, whereas AUPR (baseline 0.57) served as a stringent confirmation of top-ranked genes. We utilized R software (4.2.1) and relevant clusteProfiler [4.4.4] packages to conduct GO and KEGG pathway enrichment analyses, identifying the biological pathways and processes affected by these targets.

### 4.5. Molecular Docking Simulation

Molecular docking was performed with AutoDock Vina (1.2.x) [29] to assess the binding affinity of the four monosaccharide constituents of LVFP (arabinose, galactose, rhamnose, and glucose) toward the ten hub proteins. The crystal structures of the ten hub proteins were retrieved from the RCSB Protein Data Bank (PDB IDs listed in Figure 8). Water molecules were removed, polar hydrogens were added, and Gasteiger charges were assigned using Auto Dock Tools. The ligand structures were constructed with Chem Draw 21, energy-minimized with Chem3D (MM2 force field), and converted to PDBQT format. For each target, a grid box centered on the reported binding pocket was defined, and docking was run with default exhaustiveness and nine output poses per ligand. The lowest-energy pose was taken as the optimal binding mode, and binding energies ≤ −5.0 kcal/mol were considered favorable predicted interactions. Docked complexes were visualized with PyMOL (2.5.4) to inspect hydrogen bonds and hydrophobic contacts (Figure 8); the full binding-energy matrix is given in Table 1. No re-docking or RMSD-based validation of the docking protocol was performed; the docking results are interpreted as exploratory predictions of the binding modes of the free monosaccharides and are used only to prioritize targets for experimental validation.

### 4.6. Statistical Analysis

The data will be presented as the mean accompanied by the standard deviation (SD). Statistical evaluations will be conducted utilizing prism software 7.0. Data evaluation will involve a one-way ANOVA, followed by Tukey’s post hoc test for pairwise comparisons the variations among the groups. The *p*-value less than 0.05 will be regarded as statistically significant.

## 5. Conclusions

In conclusion, we demonstrated that LVFP exerts significant hepatoprotective effects on ALI in vivo. Integrated network pharmacology and ROC-PR-informed transcriptomic analysis propose a VEGFR2/PKM2-centered working hypothesis, which awaits direct protein-level validation in future studies. This study provides pharmacological evidence for LVFP and proposes a refined target list for future mechanistic dissection.

## Figures and Tables

**Table 1:** Molecular docking energies of the monosaccharide constituents of LVFP with the hub anti-ALI target proteins.

No.	Target Protein (PDB ID)	Ligand (PubChem CID)	Docking Energy (kcal/mol)
A	SRC (1NZL)	Arabinose (4319195)	−6.45
B	HSP90AA1 (5NJX)	Arabinose (4319195)	−5.58
C	HSP90AA1 (5NJX)	Galactose (6036)	−5.92
D	GSK3β (2O5K)	Galactose (6036)	−7.68
E	AKT1 (1UNP)	Rhamnose (25310)	−7.36
F	ENO1 (7V67)	Glucose (5793)	−5.40
G	ENO1 (7V67)	Galactose (6036)	−5.37
H	HSP90AA1 (5NJX)	Rhamnose (25310)	−6.47
I	HSP90AA1 (5NJX)	Glucose (5793)	−5.57
J	VEGFR2 (3WZE)	Rhamnose (25310)	−8.65
K	TPI1 (4ZVJ)	Rhamnose (25310)	−5.82
L	PKM2 (7R6Y)	Rhamnose (25310)	−5.74
M	GPI (8BBH)	Galactose (6036)	−6.64
N	TPI1 (4ZVJ)	Galactose (6036)	−5.69
O	GPI (8BBH)	Arabinose (4319195)	−5.58
P	GPI (8BBH)	Glucose (5793)	−5.36
Q	GPI (8BBH)	Rhamnose (25310)	−6.84

## Data Availability

The original contributions presented in this study are included in the article/Supplementary Materials. Further inquiries can be directed to the corresponding authors. The study analyzed publicly accessible transcriptomic data. The data can be found here: https://www.ncbi.nlm.nih.gov/geo (accessed on 2 August 2024); accession number GSE217659.

## Supplementary Materials

The following supporting information can be downloaded at: https://www.mdpi.com/article/10.3390/ijms27198805/s1.
