# Supplementary material for: Ligustrum × vicaryi Fruit Polysaccharide Attenuates LPS/D-GalN-Induced Acute Liver Injury in Mice
Source: Int J Mol Sci. 2026 Oct 1;27(19):8805. doi: 10.3390/ijms27198805 (PMC13664983; doi:10.3390/ijms27198805)
Supplement: Supplementary file 1 [file ijms-27-08805-s001.zip › ijms-4513041-supplementary.pdf]

Figure S1. Glucose standard curve for the anthrone–sulfuric acid assay ($y = 7.67x - 0.0181$, $R^2 = 0.9997$).

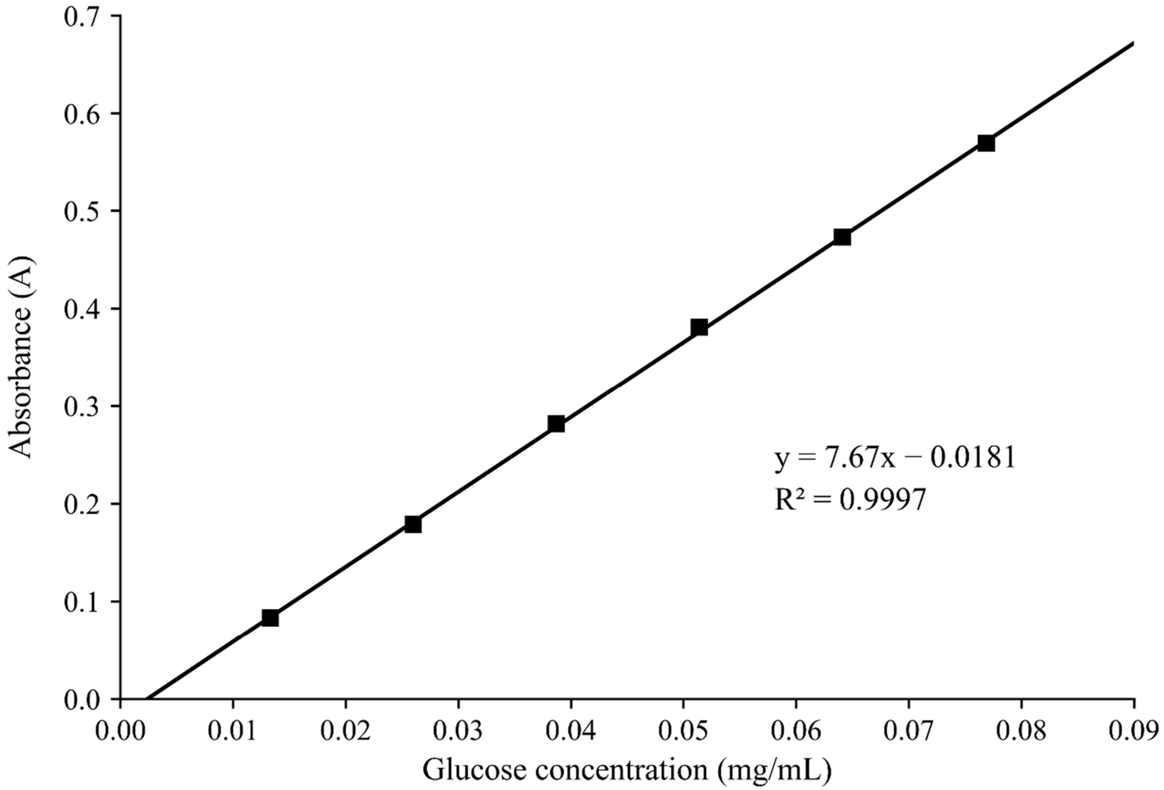

Figure S2. Structural characterization of the *Ligustrum vicaryi* L. fruit polysaccharide (LVFP). Fourier transform infrared (FTIR) spectrum of the LVFP, showing characteristic absorption bands at 3345 (O–H stretching), 2929 (C–H stretching), 1599, 1412, 1281, and 1073 cm^{-1} (C–O/C–O–C stretching).

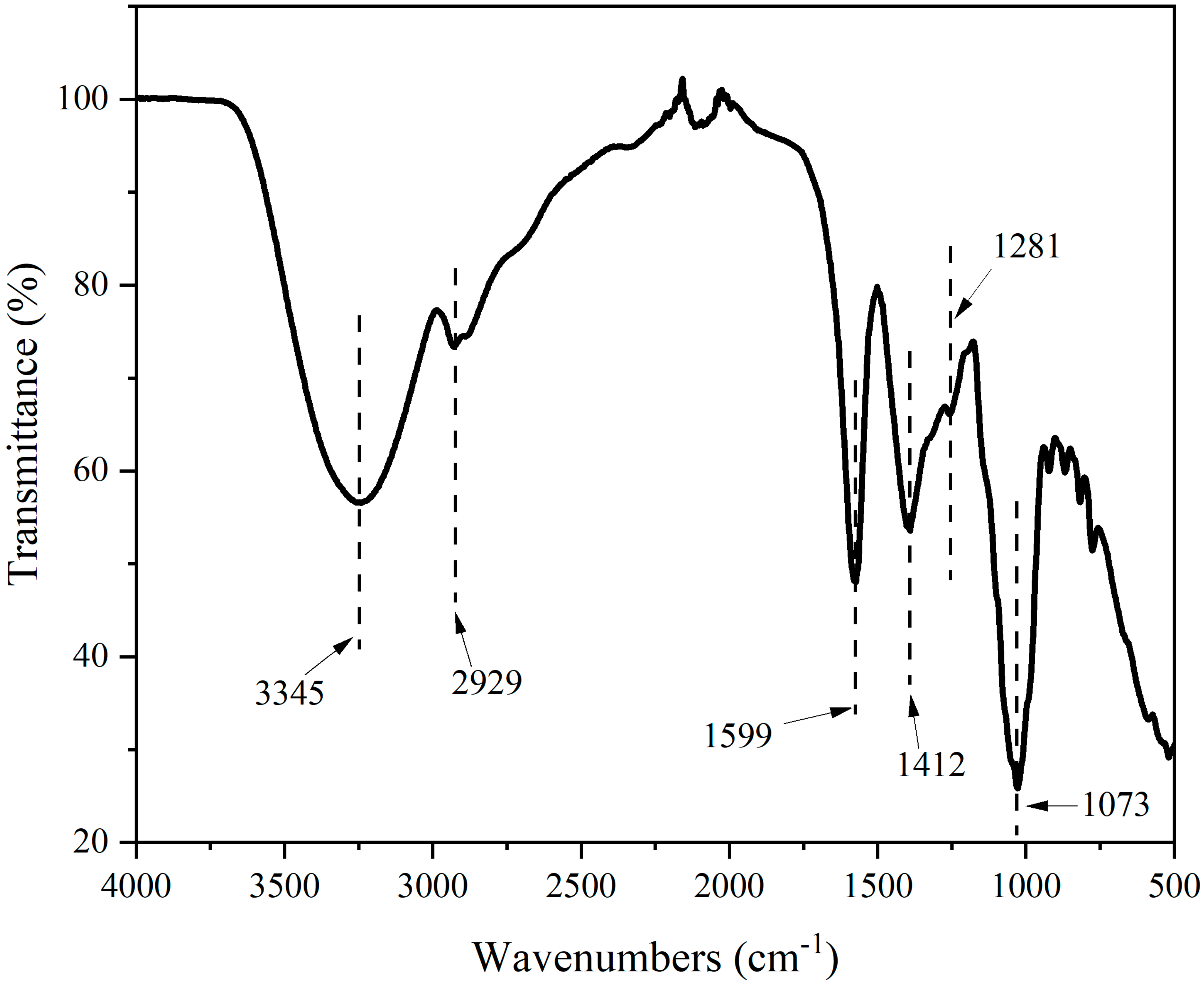

Transmittance (%)
100
80
60
40
20
4000
3500
3000
2500
2000
1500
1000
500
Wavenumbers (cm-1)
3345
2929
1599
1412
1281
1073
